# Track Thyself? The Value and Ethics of Self-knowledge Through Technology

**DOI:** 10.1007/s13347-024-00704-4

**Published:** 2024-01-27

**Authors:** Muriel Leuenberger

**Affiliations:** Center for Ethics, University of Zurich, Zollikerstrasse 117, 8008 Zurich, Switzerland

**Keywords:** Self-knowledge, Bioinformation, Algorithmic profiling, Relational identity, Ethics of self-knowledge

## Abstract

Novel technological devices, applications, and algorithms can provide us with a vast amount of personal information about ourselves. Given that we have ethical and practical reasons to pursue self-knowledge, should we use technology to increase our self-knowledge? And which ethical issues arise from the pursuit of technologically sourced self-knowledge? In this paper, I explore these questions in relation to bioinformation technologies (health and activity trackers, DTC genetic testing, and DTC neurotechnologies) and algorithmic profiling used for recommender systems, targeted advertising, and technologically supported decision-making. First, I distinguish between impersonal, critical, and relational self-knowledge. Relational self-knowledge is a so far neglected dimension of self-knowledge which is introduced in this paper. Next, I investigate the contribution of these technologies to the three types of self-knowledge and uncover the connected ethical concerns. Technology can provide a lot of impersonal self-knowledge, but we should focus on the quality of the information which tends to be particularly insufficient for marginalized groups. In terms of critical self-knowledge, the nature of technologically sourced personal information typically impedes critical engagement. The value of relational self-knowledge speaks in favour of transparency of information technology, notably for algorithms that are involved in decision-making about individuals. Moreover, bioinformation technologies and digital profiling shape the concepts and norms that define us. We should ensure they not only serve commercial interests but our identity and self-knowledge interests.

## Introduction

Novel and emerging technologies promise us unique insights into who we are. Technological devices and applications measure, label, categorize, and diagnose us. In this paper, I explore the value and ethics of self-knowledge through technology. If we have practical and moral reasons to pursue self-knowledge, and those technologies promise us more of it – should we use them to increase our self-knowledge? To address this question, we need a better understanding of the kinds and quality of self-knowledge they provide. I distinguish between three different kinds of self-knowledge (impersonal, critical, and relational) that give us different types of insights into who we are, investigate how technology can contribute to them, and raise ethical issues with technologically sourced self-knowledge.

I focus on technologies that provide bioinformation – specifically, health and activity trackers, Direct-to-Consumer (DTC) genetic testing, and DTC neurotechnologies – as well as algorithmic profiling used in recommender systems, for targeted advertising, and to support decision-making in areas such as the justice system, health care or the job market. Bioinformation technologies and algorithmic profiling have both been specifically designed to characterize us and are presumably the biggest sources of technologically sourced personal information. Moreover, they represent two different ways how self-knowledge can be conveyed to us. Bioinformation technologies usually aim at providing personal information about the user to the user themself (of course, companies often collect and use this data as well). The personal profiles generated by algorithms are typically used to alter our behavior and support decision-making about us. In most cases, they reach us and potentially contribute to our self-knowledge only indirectly, through advertisements, recommendations, and decisions about us. One of the central ethical concerns that arise for bioinformation technologies and algorithmic profiling are privacy issues. In this paper, privacy concerns are largely set aside, to argue that ethical challenges arise not only when personal information gets into the wrong hands but also when it gets into the hands of the individuals themselves.

In philosophy, self-knowledge tends to be discussed in epistemology, not in ethics.^1^ The focus is on self-knowledge as knowledge of one’s mental states (e.g., knowing that you feel cold) and the question whether self-knowledge differs from our knowledge of the external world (Gertler, 2021). However, to investigate the value and ethics of self-knowledge through technology, a broader concept of self-knowledge is required. The morally and practically valuable self-knowledge is *substantial* knowledge (see Section 3.2) about *who one is*. This includes mental states as well as physical properties and, as I argue, how we are defined by others. While we can observe a relational turn in the literature on the self and identity which acknowledges that the self is at least partially defined by and through others (Baylis, 2012; Lindemann, 2001; C. Mackenzie & Stoljar, 2000; Wallace, 2019), there is no corresponding debate on relational dimensions of self-knowledge. This paper argues for relational self-knowledge as a distinct kind of self-knowledge and explores the implications of technologically sourced self-knowledge for relational self-knowledge. Thereby, it helps to broaden our understanding of the scope of self-knowledge technology can provide. Relational self-knowledge is an important source for knowing oneself to which bioinformation technology and algorithmic profiling can contribute, given the right circumstances.

The literature concerned with self-knowledge through technology primarily explores self-tracking technology and largely considers as self-evident that technology can contribute to self-knowledge and mediate self-reflection (Dietrich & Van Laerhoven, 2016; Lanzing, 2016; Lupton, 2016). The promise of self-knowledge is often a motivating factor for buying and using this technology – the slogan of the Quantified Self Movement is *self-knowledge through numbers* – and a major element in the advertisement of self-tracking technologies.^2^ But what *kind* of self-knowledge those technologies provide and whether they contribute valuable and substantial self-knowledge which we have moral and practical reasons to pursue has so far not been sufficiently explored. Moreover, the paper broadens the scope of technologies that can contribute to self-knowledge by including considerations on digital profiling.

To investigate the value and ethics of self-knowledge through technology, I proceed in three steps. First, I introduce bioinformation technologies and algorithmic profiling with a focus on what personal information we may gather through them. Second, the notion of self-knowledge is explored by examining the value of self-knowledge, considering which forces define and shape the self, and distinguishing between three types of self-knowledge (impersonal, critical, and relational). Third, I analyze the value and ethics of self-knowledge through technology on the basis of the three types of self-knowledge distinguished earlier. In this section, I discuss how technology can contribute to the three types of self-knowledge, the quality of self-knowledge it provides, and which ethical issues arise in the pursuit of self-knowledge through technology.

## Self-knowledge through technology

In the last decades, technological devices and applications have been produced that can measure, collect, and infer a great deal of personal information. In the following, I focus on two types of technologically sourced self-knowledge: 1) bioinformation – information about one’s body generated by technological devices and applications and 2) algorithmic profiling – the categorization and characterization of individuals for recommender systems, targeted advertising, and similar applications as well as to support decision-making about individuals. Both technological bioinformation and algorithmic profiling have become much more widespread and accessible in the last decade. In the following, I briefly discuss those technologies and what they can potentially or allegedly tell us about ourselves.

1. In terms of bioinformation, three technologies are particularly relevant for providing accessible, large amounts, and potentially substantial personal information: health and activity trackers, DTC genetic testing, and DTC neurotechnologies. They can potentially provide novel information about us, confirm self-knowledge we already had on another basis (measured, neuronal, genetic), or help us to change ourselves.
  Health and activity trackers are a rapidly growing field of technological bioinformation. A health tracker can range from a basic menstruation calendar to a sophisticated device that monitors a vast range of physiological metrics such as heart rate, sleep cycles, temperature, and blood oxygen levels. Health tracking devices or applications are commonly used to improve fitness, for weight loss, or to monitor medical conditions (Lupton, 2016). DTC genetic testing provides further technological access to personal bioinformation. Genetic testing promises to diagnose or rule out genetic disorders or predict the risk for some conditions, to determine biological relatives (most commonly to determine who is the biological father of a child), to determine ancestry, and to provide information on personality, athletic ability, or child talent. However, they often report false positives and false negatives and are prone to misinterpretations (Horton et al., 2019). The price for genetic sequencing has decreased drastically since the mapping of the human genome in 2003. This has led to a spike in genetic testing (Phillips et al., 2018). DTC neurotechnology claims to deliver unique insights into body and mind, read emotional states and stress levels, and improve sleep, focus, cognitive and athletic performance, memory, relaxation, and more. It typically employs EEGs, but galvanic skin responses, electroshocks, or tDCS are also used to record and modulate the brain (Ienca & Vayena, 2019). Many of the promises made by DTC neurotechnology companies are untested and likely exaggerated (Wexler & Reiner, 2019).
2. Algorithmic profiling can be used to draw far-reaching inferences about an individual’s characteristics, beliefs, desires, and behavior from online traces and other databases. Age, gender, sexual orientation, race, employment status, political opinions, preferences in food, clothes, news, entertainment, and other products, or whether one is likely to have insomnia or depression can be inferred from social media posts, search histories, traces we leave online, and data collected by personal digital devices (Huckvale et al., 2019; Loi, 2019). This algorithmic characterization is typically fluid – we move in and out of categories based on our most recent online behavior (Prey, 2018). An algorithmic profile can be very simple and contain only a small number of data points (e.g., a user profile at a pizza delivery store) or it can be elaborate and detailed, such as the user profiles generated by Google or Facebook that not only collect personal information across different platforms but create and hunt user data (Véliz, 2020). Furthermore, algorithmic profiling is employed to make decisions that can profoundly impact individuals. It is, for instance, already implemented in job recruiting and selection, in medical decision-making, and in the justice system (Čartolovni et al., 2022;﻿ Hunkenschroer & Luetge, 2022; Završnik, 2020). Algorithmic profiling is used to decide which jobs are advertised to you on LinkedIn or offered in public employment services, whether your application makes it to the next round, the type of health care allocated to you, whether you are considered to have a low recidivism risk so you can get reduced sentencing or bail, or whether you get precautionary police surveillance.

For this technologically sourced personal information to become self-knowledge it needs to reach the individual himself or herself. This can be done explicitly, as in health trackers, or DTC neuro- and gene technologies, where individuals receive direct access to the measured personal information. Or this information can reach the individual implicitly, for instance by showing ads and recommendations, by triggering a pop-up with information about mental health support, or by being involved in decision-making processes about the individual. Some algorithmic profiling companies offer both. For instance, Google shows you personalized ads based on your user profile, but you can also look up how they characterize you (this is only possible if you have targeted advertising activated).^3^ Of course, a lot more information about us is collected and inferred which never reaches us, either directly or indirectly, in a way that could expand our self-knowledge, such as information collected in some research contexts, or information used to aggregate user behavior to adjust a homepage design.

Both bioinformation and algorithmic profiling provide an external perspective on us. This in itself is not unusual. Other people routinely provide feedback on who they take us to be and even objects can provide such an external perspective – clothes give feedback on changes in body size, or voice recognition on how understandable your speech is. But those novel technologies for tracking bioinformation and algorithmic profiling are *designed* to characterize you and give feedback on who you are – either to yourself or to a company (and then indirectly back to you). Bioinformation technologies provide an external perspective that is primarily focused on physical properties, whereas algorithmic profiling typically engages with concepts shaped by cultural practices, institutions, or artefacts such as race, being a large employer, a likely reoffender, or an SUV enthusiast.

## Self-knowledge

### The self

To understand what self-knowledge encompasses and how technology can contribute to self-knowledge, it is helpful to look at which forces contribute to defining and shaping the self. As I argue in the following, the self can be understood as discovered, actively created, as well as defined and shaped through relations with others. Those three dimensions that together construe and define the self also delineate the realm of self-knowledge.

The self is partially a matter of self-discovery (Levy, 2011; Parens, 2005). There are some “hard facts” about yourself you may discover, for instance, where and when you were born or how tall you are. Those aspects of the self, which Sartre labelled “facticity” (Sartre, 1956), provide the backdrop within which human freedom exists and is constrained. Besides brute facts about our personal history and physical properties, some beliefs, values, goals, or traits can also appear to be discovered rather than chosen. There are things about us that seem particularly fitting to who we are and that might seem hard or nearly impossible to change. You might discover that you are very timid and that there is not much you can do about it.

But to some degree, you can also create yourself (DeGrazia, 2005; Levy, 2011; Parens, 2005). You can decide if you want to be a philosopher or a boxer, whether you help your friends when they ask you to, or whether you try to be more outgoing. Through choices and actions, we create facts about ourselves and are actively involved in constituting ourselves. Additionally, we define ourselves through interpretation and evaluation (Glover, 1988; MacIntyre, 1984; Sartre, 1956). We interpret and evaluate our actions, as well as the “hard facts” about ourselves, and decide what they mean for us – how they feature in our self-concept and influence future actions, goals, and plans. For someone, being French might be an important part of their identity while for another person this same fact about their heritage is largely irrelevant to who they are and how they plan and lead their life. Such interpretations and evaluations of oneself can change over time.

Self-discovery and self-creation do not occur in isolation. We are socially embedded in relationships, communities, and cultures that shape and constrain our beliefs, desires, goals, values, actions, and ultimately, our identity. We are interconnected with others and define ourselves with, through, and in opposition to others (Baylis, 2012; Butler, 2001; Fivush et al., 2011; Lindemann, 2001; Taylor, 1989). Others shape our selves in *indirect* and *direct* ways. The former refers to influences whose potential to define who we are depends a great deal on how we respond and engage with them. We can reject, resist, ignore, or embrace those indirect influences. Whether and to what degree we are defined by them (in the sense that they influence our behavior, values, beliefs, self-conception, etc.) is to a degree up to us. Others shape us indirectly through concepts and norms we acquire in a social context that impact how we think about the world and ourselves (e.g., what do labels like liberal, black, horror-movie buff, or lazy mean and do they apply to me?), they suggest which self-descriptions are appropriate for us individually and as members of a group, they function as examples and comparisons, we learn to know ourselves by interacting with and comparing ourselves to others, and we regularly have to give an account of ourselves and our actions to others and define ourselves more or less intelligibly in the course of doing so. We can resist or reject those influences, for instance by reinventing and altering the available concepts to describe ourselves. However, a common cultural ground remains the starting point for such projects of self-definition.

Others also define us through our *relations* to them. Some of those relations we can pick ourselves, like friendships or being a member of a book club, and depend to some degree on the cooperation of the other(s) in that relation. Other relations are largely given, such as biological parents or membership of an ethnic group. Nonetheless, we can influence what those relationships mean for ourselves and to what degree they define us (is being part of this ethnicity an important and self-defining or a largely irrelevant aspect of my identity?).

But there are also more incisive, direct ways in which others define us. Notably, others define us through (often physical) *constraints and opportunities*. They can grant or deny access to places and institutions and offer opportunities for jobs, events, or connections. Others can strongly influence one's own scope of action and offer or restrict possibilities for action. Insofar as we are defined by our actions, others define us by shaping our scope of action. In a similar vein, others can impact and alter our bodies which are also defining us. Moreover, our bodies, actions, and the environments we are exposed to can indirectly shape our values, beliefs, interests, and capabilities (e.g., daily repeated actions and environments at work or a traumatic physical injury can change goals, beliefs, and values). We can relate to these constraints or opportunities in different ways, thereby choosing what they mean for us and how much we let them define us, and we can try to change or fight them. But to some degree, it is up to others who we can be.

Some might argue that such constraints and opportunities are not *truly* defining us. A woman in Afghanistan who cannot go to university still has the intelligence and other capabilities to study. The admission rules to the university change nothing about that – they change nothing about *her*. But we are defined by more than our potential, aspirations, and values. What *you actually end up doing* in your life matters greatly for who you are. Either you are a doctor who dedicates her life to her patients and finds meaning in doing so or you are not. The self is not what is somehow “behind” or “beyond” our messy personal, sociocultural circumstances or who we could have been if we grew up and lived in optimal open and nurturing conditions, whatever those might be. It is who you are in the midst of your particular circumstances, including both potential, values, intentions, and aspirations as well as actions and physical properties. Some of those self-defining characteristics can be shaped and changed by others. Just as we can create facts on the matter of who we are, others can create facts on the matter of who we can be.

Self-discovery and self-creation can both apply to the relational aspect of the self. We can discover, for example, how others relate to us and define us and we can make choices, for instance, about who we interact with, how we relate to others, and what our relations to others or how they define us mean for us. The self is objective (an object of inquiry and discovery for ourselves and others), subjective (experienced and shaped through a subjective first-personal perspective and agency), and relational (influenced and constrained by others).

Let us consider the example of race to illustrate the interplay of the three dimensions. Some hard facts about ancestry and skin pigmentation are relevant for whether someone is, for instance, white. But the self is also defined by what being white means for this person. Is it a feature they do not care much about or one they deeply identify with and that strongly influences their values and beliefs, as, for instance, in the case of a white supremacist? But of course, race also has many social connotations. What being of a certain race means depends on social structures, institutions, and meanings. Others can also decide whether they let you define yourself as being of a certain race (see, for instance, the racial classifications of apartheid South Africa). How and to what degree race defines someone depends highly on whether they live in a racist society, how others judge their race, and which culture and stereotypes are connected to a race.

### Self-knowledge: impersonal, critical, and relational

Before looking into how those three dimensions of the self correspond to three types of self-knowledge, I want to briefly discuss the value of self-knowledge. Why should we make an effort to at least overcome some of our self-ignorance? We can distinguish (at least) four reasons. 1) Self-regarding usefulness: self-knowledge has straightforward personal advantages.^4^ It can improve your well-being because it puts you in a position where you can predict, control, and explain yourself and make better plans and decisions. 2) Other-regarding reasons: knowing yourself helps others in multiple ways. If you know yourself well you are in a better position to be a reliable co-operator, cost the health system less and you may be more virtuous and morally good because you are less deceived about your true motives and weaknesses (Kant, 2011; O'Hagan, 2017). 3) High-road reasons: it has been argued that self-knowledge supports or is necessary for a range of abstract, high-sounding ideals (Cassam, 2014), such as for critical reasoning (Burge, 1996; Shoemaker, 1988), responsibility (Moran, 2001; Tugendhat, 1986), autonomous self-governance (Meyers, 2004), and authenticity (Leuenberger, 2020). Duty to know oneself: finally, it has been argued that you owe it to yourself to know yourself (Kant, 2011; J. Mackenzie, 2018). For Kant, this duty to oneself grounds all moral duties. It mitigates the tendency to deceive yourself into believing that what you want is morally right.

Insofar as self-knowledge is instrumentally valuable – it is valuable because it contributes to well-being, virtues, a duty to self, responsibility, authenticity, etc. – more self-knowledge is not always better. A constant, self-obsessed pursuit of self-knowledge is counterproductive to reach the goods self-knowledge promises. Investing too much time and energy in acquiring self-knowledge means that you may miss out on doing other things conducive to your well-being or your moral development, you might be neglecting or annoying others by constantly focusing on yourself, you could fail to commit to or follow through with other projects in your life that may be important for your authenticity and autonomy, and a duty to self likely does not demand that one should know everything about oneself there is to know. Thus, there is not just a shrinking return on investment for an extensive pursuit of self-knowledge, but it can have negative consequences. Self-knowledge might also be intrinsically valuable. But even in that case, it seems plausible that only meaningful, substantial insights are relevant for intrinsically valuable self-knowledge and that an excessive pursuit of self-knowledge would conflict with other intrinsically and instrumentally valuable goods. Thus, the value of self-knowledge does not imply that we should pursue it at all costs.

The goods self-knowledge promises require not just any kind of knowledge. Knowing how many hairs you have on your head or that you wear socks is not particularly useful to others or yourself and it does not suffice to fulfill a duty to self or ground high-road ideals. The kind of self-knowledge we need is more substantial.^5^ “Just as you can learn your best friend’s blood type without feeling that you have learned anything about her, so, too, can you learn that you put your left sock on first this morning without feeling that you know yourself any better as a result.” (J. Mackenzie, 2018, 259).

Cassam provides an account of what substantial self-knowledge entails (Cassam, 2014). Roughly speaking, substantial self-knowledge is what defines you as a person: knowledge of your character, values, abilities, aptitudes, emotions, and what makes you happy. Examples of substantial self-knowledge are knowing that you are timid, not a racist, in love, able to do a statistical analysis, that changing your career would make you happy, or why you believe the earth is flat. Substantial self-knowledge requires effort, is fallible, can be challenged and corrected, is based on evidence, it is relevant to your self-conception, and it matters in a practical or moral sense. Those characteristics of substantial self-knowledge are not meant as necessary and sufficient conditions, but they can give us a rough indication for relevant considerations to determine whether a kind of self-knowledge is substantial. The distinction between substantial and trivial self-knowledge is a matter of degree and the more of these characteristics a piece of self-knowledge has, the more substantial it is (Cassam, 2014, 29–30).

In light of the three dimensions of the self, I suggest understanding self-knowledge as involving three corresponding elements: impersonal, critical, and relational self-knowledge. Knowing yourself means knowing hard facts about yourself, knowing who you decide to be, and knowing how you relate to others. For the distinction between impersonal and critical self-knowledge, I rely on a paper by Neil Levy (Levy, 2016). The relational dimension has so far been neglected in the debate on self-knowledge. The relational turn in the literature on identity and the self seems to not have reached the debate on self-knowledge yet.

Impersonal self-knowledge covers facts about yourself, independent of your agency and what you think about them. It is knowledge about you that is gathered by the same or analogous methods you use to know other people (Levy, 2016). This includes inferring your character or beliefs by observing patterns in your behavior, asking others what they think about you, or when you use technologies to measure and represent you, such as a brain scan or a health tracker. It is knowledge about how you appear to an external perspective, from an objective (in contrast to a first-personal, subjective) viewpoint. This type of self-knowledge is concerned with the dimensions of the self we can discover.

The process of gaining impersonal self-knowledge can be very elaborate and active, but the individual remains a somewhat passive recipient of information about who they are instead of an active creator. For instance, Mira thought she does not like hiking but after going on her first hike she realizes that she loves it. The joy she felt when she reached the top convinces her that she was wrong in her assumption that she dislikes hiking. In this example, Mira is passive regarding the content of the phenomenological experiences that inform her about her likes and dislikes (even though she might deliberately seek out those experiences).

Critical self-knowledge, on the other hand, is agential, an essentially practical ability (Levy, 2016). The content of critical self-knowledge is not just discovered by the individual but actively generated. You are the author of those beliefs, attitudes, and intentions. They do not just occur to you, but you exercise agency over them. Critical self-knowledge is acquired by looking to the world and deciding what you have reason to believe and desire, in contrast to looking inward and finding out what you happen to believe or desire. In doing so, you constitute yourself and take responsibility for your mental states. “One is an agent with respect to one’s attitudes insofar as one orients oneself toward the question of one’s beliefs by reflecting on what’s true, or orients oneself toward the question of one’s desires by reflecting on what’s worthwhile or diverting or satisfying.” (Moran, 2001, 64) We can be active and take responsibility for our mental states because we can avow, change, or reject them. We alter ourselves by interpreting our mental states, deciding what to believe or do, and by forming our desires, thoughts, and feelings (Moran, 2001). “Self-knowledge crucially is also a matter of avowal, of *making* true what is claimed to be true by acting on the balance of reasons that present themselves in the scenario at hand” (Bransen 2015, 316). This is self-knowledge from the perspective of a first-personal agent who can make up their mind about and take a stance on their mental states. This critical self-knowledge ties in with dimensions of the self that are actively and dynamically created.

Critical self-knowledge can be applied to impersonal self-knowledge. We can actively engage with impersonal self-knowledge and interpret, embrace, ignore, or reject it. Figuring out what the hard facts you discovered about yourself mean for your identity is a matter of interpretation, and to an extent, that interpretation is up to you (Leuenberger, forthcoming-a). This interpretation does not leave their objects unaltered (Moran, 2001). Mira might not just passively learn that she likes hiking but ponder whether she has good reasons for liking it, actively embrace this part of herself, make new plans and resolutions based on it, and give it her own meaning. Critical and impersonal self-knowledge are often intertwined.

Finally, self-knowledge is also knowledge about how you fit into the world. This encompasses knowledge about other’s direct influences on your practical realities, such as which groups or institutions you belong to, which opportunities and freedoms you have, and which restrictions you face. This direct relational self-knowledge can be of great practical value. It informs you about your scope of action and who others will let you be. Relational self-knowledge concerning other’s indirect influences on you is less tangible. It could, for instance, encompass knowledge about concepts and norms that are being applied to you. Self-knowledge involves interpretation, evaluation, and organization of information, finding patterns, connecting actions to overarching goals or dispositions, labelling, and more. This process is guided by socially shared and often disputed concepts – and technology is involved in negotiating those concepts. Knowing yourself can entail knowing how others (including technology) represent you, which labels they apply, and what those labels mean because they can shape your self. “Who we are reflects how it is possible to talk about us, which categories are in use, how the categories are employed, and what they mean, as well as how data are used to represent us in databases. At the same time, categories and formats have an influence on who we can be.” (Søe & Mai, 2022).

Who you take yourself to be influences how others see you and whoever they take you to be and allow you to be influences how you (can) see yourself. Self-knowledge includes knowing how you are seen by others because they can enable and limit which identities you can enact and conceptualize. This relational self-knowledge combines features of impersonal and critical self-knowledge. It is knowledge you *learn* about instead of knowledge you *create*, just like impersonal self-knowledge. However, it nonetheless involves active creation of a self, albeit by others. Relational self-knowledge is not about learning independent facts about yourself (as in impersonal self-knowledge) or creating yourself (as in critical self-knowledge) but about learning how others co-create you. There can be some overlap between impersonal and relational self-knowledge. For instance, someone telling you that you are too introverted for a job contains impersonal self-knowledge about a character trait of yours as well as relational self-knowledge about opportunities others offer or deny you based on your characteristics.

Both you and others can be wrong about your identity. It is a matter of dispute who has ultimate authority about who you truly are and there may be no objective truth on the matter. But both the first-personal and the external perspectives constitute and shape distinct practical realities that result from the constructed identities. Your self-conception will influence how you perceive the world and how you act, choose, and plan your life. The other’s conception of you influences who they let you be, which conceptual resources they provide you with to define yourself, and what kind of opportunities, freedoms, and constraints you have. Because both perspectives define you, in the sense that they define how you perceive yourself and the world as well as how you (can) act in it, knowing yourself entails knowing both spheres – the internal and the external. When we are concerned with practically and morally valuable self-knowledge, such a broad notion of self-knowledge seems particularly relevant.

Usually, knowledge is concerned with information that is already there and determined. When you learn about the laws of gravity, you get access to information that existed independently of anyone knowing it. Self-knowledge is different. There are aspects about you that are not determined yet. When you engage in practices of critical self-knowledge, you do not *access* existing information but instead *create* facts of the matter. But in the end, you will know more about who you are – you increased your self-knowledge. And just like you create facts about yourself, others can create facts about you. Coming to know them means coming to know yourself.

## Value and ethics of self-knowledge through technology

Given that technology can provide vast amounts of personal information and that we have practical and moral reasons to pursue self-knowledge – should we learn to know ourselves through technology? As I argue in the following, (4.1) the quality of the personal information is central and often not ensured by technologically sourced self-knowledge, particularly for marginalized groups, (4.2) technology provides impersonal self-knowledge of a kind that makes critical engagement particularly difficult, (4.3) but because we are shaped, defined, and restricted by technology on a relational level with potentially significant ramifications, knowing how technology characterizes us can be an important piece of self-knowledge – even if it does so inaccurately.

### Impersonal self-knowledge: quality over quantity

The technologies introduced earlier can make claims about substantial characteristics. Health and activity trackers let you know more about your abilities and aptitudes, DTC genetic testing can inform you about a genetic disorder that will limit future opportunities, DTC neurotechnologies provide information about emotional states and stress levels, and algorithmic profiling is used to infer interests, preferences, character traits, skills, beliefs, and future behavior. However, algorithmic profiling tends to be less likely to reveal substantially new information about yourself compared to bioinformation technologies. The latter are designed to provide the user with novel information about their bodies. The algorithmic profiles should inform companies and institutions about the individual who have little to no prior knowledge about them, except for, for instance, login data. The information algorithmic profiling is typically after, such as someone’s political affiliations, religious beliefs, or hobbies, are already known to the individuals themselves and thus tend to bear less potential for relevant self-knowledge. Moreover, due to privacy concerns, many users are obfuscating their data with the aim that data mining companies cannot generate a truthful profile (Brunton & Nissenbaum 2015) which means that their profiles are no reliable sources for self-knowledge.^6^

But both bioinformation technology and algorithmic profiling can provide information through which one can gain substantial self-knowledge by reflecting on it or ascribing meaning to it. Information about someone’s genetic ancestry is in and of itself not necessarily substantial self-knowledge but many people who use services like 23andMe (one of the largest providers of DTC genetic testing) identify with their heritage and ascribe personal meaning to it. Being, for example, 25% Croatian becomes an important part of who they are. Getting categorized by Google as an animal lover or as a much older person than you really are might spur some reflection on why its algorithms come to this conclusion and whether this is true, which could lead to meaningful personal insights.

Even in cases where technology just confirms what you already knew (e.g., even without a health tracker, you already know that you sleep very poorly), personal information technology can give you biochemical, measured, quantifiable data on what you already knew. Thereby, it can provide further justification for beliefs about yourself. As we know from epistemology, true beliefs are not knowledge – they require *justification*. Technology can, in some cases, justify your beliefs about yourself or make them more justified.

In the best case, biotechnologies and algorithmic profiling can broaden self-knowledge and help to overcome self-deception and self-ignorance. They can help you to get a clearer and richer picture of who you are. If the provided information is reliable and substantial, technology can provide you with all the benefits of self-knowledge – from better decision-making to supporting others, being more virtuous, fulfilling a duty to yourself, and potentially even to increase agency, freedom, and responsibility.

But technology providing substantial, reliable, and accurate personal information is not the standard situation. Technology is often inaccurate or biased. The highly unregulated market of DTC neurotechnologies, for instance, has led to vast quality differences and there is little scientific evidence that the devices deliver what they promise (Wexler & Reiner, 2019). If you look up how Google characterizes you, you will probably agree that while they get many things eerily right, there are also some obvious mistakes. And algorithmic profiling employed in the justice system and job market has repeatedly proven to be biased, e.g., for race and gender (Mann & Matzner, 2019). Personal information technologies also provide a lot of trivial information. For instance, the constant measurement of a vast range of physical parameters by some health trackers floods users with trivial information about detailed aspects of their bodies. Many digital profiles created about users are only based on a single or a few purchases and offer very little substantial information (e.g., the user profile of a pizza delivery store).

If technology provides flawed information and characterizations, it threatens self-knowledge and its benefits. It can lead to a misleading self-conception and flawed decision-making by and about the individual. Technology can also render users insecure in their self-knowledge if it contradicts other sources of self-knowledge. However, when considering the quest for self-knowledge through technology, it is important to compare it with the other imperfect methods of self-discovery available to us, rather than an unattainable ideal. In some cases, even flawed technology will be better than what we have. Many of the “simpler” bioinformation technologies, such as devices that measure one’s heart rate, temperature, or sleep cycle, likely meet this threshold. DTC neurotechnologies and algorithmic profiling, on the other hand, probably contribute less to impersonal self-knowledge than a conversation with a friend or honest introspection would. Besides accuracy, we can compare the quality of self-knowledge regarding its depth or specificity. Technology often makes more specific claims than other means of acquiring self-knowledge (e.g., your stress levels rose by 23% within the last 20 min compared to you feeling a bit nervous). But specificity tends to come at the cost of accuracy. This connection can make evaluations regarding the quality of self-knowledge difficult. How does rightly believing that your ancestors are from Europe compare to falsely believing they are from Northern Ireland when they are in fact from the south of Ireland? Such questions depend on the specific case as well as on the value of self-knowledge that is pursued.

The benefits of self-knowledge can also be threatened by an obsessive engagement with personal information technologies. Algorithmic profiling is less problematic in this regard since the information in those personal profiles reaches the individuals only indirectly and can often not be accessed or generated at will. But biotechnologies are often designed such that the user is nudged to continually engage with the technology. In some cases, the extensive investment of time and energy in the pursuit of self-knowledge is no longer conducive to the goods self-knowledge can provide. Moreover, such technologies may gradually limit our abilities for introspection and intuitive self-knowledge. If a DTC neurotechnology device or an algorithm can inform me about my stress levels and emotional states, why should I pay attention to it? Technology might lead to a deskilling of our abilities for acquiring self-knowledge. This might impede self-knowledge in areas where technology does not provide us any insights and in cases where personal information technology is no longer available.

Bioinformation technology and algorithmic profiling regularly disadvantage marginalized groups in terms of accuracy, bias, and how extensive and detailed personal information is. Just to give some examples, smartwatches have been found to be less effective at tracking the health of people with dark skin (Koerber et al., 2022), algorithmic predictive policing and predictions of dangerousness have been shown to be biased (Alikhademi et al., 2022; Tonry, 2019) as have hiring algorithms, and genetic ancestry testing is much more accurate in determining the geographic origin of the ancestors of people with European heritage – of the over 2000 geographic regions to which 23andMe traces ancestry, only 167 are in Africa, compared to 164 in the United Kingdom alone (23andMe, 2023). Such discrepancies in the quality of personal information can exacerbate already existing disadvantages in health, hiring processes, the justice system, and other areas for marginalized groups. However, if we again compare the information technology provides to other available, often also biased information (e.g., to the assessment of a biased doctor), even flawed technology might be more accurate in certain instances.

This significant ethical challenge of disadvantages in the quality of technological personal information for marginalized groups warrants further research that specifically analyzes those technologies as sources of self-knowledge. Open questions that should be addressed further concern the extent of this information quality gap, how this lacking quality in technological sources of self-knowledge impacts the well-being of marginalized groups across different areas, and whether and how discrimination in personal information technologies should be regulated on the grounds of self-knowledge interests.

In cases where technology can provide accurate and relevant information, we seem to have pro tanto ethical and practical reasons to pursue self-knowledge through technology. In this best-case scenario, we gain information through those technologies that can help us to make better decisions, turn us into better cooperators, fulfill a duty to ourselves, and increase our agency. For instance, bioinformation technology that increases your self-knowledge about your body and thereby helps you to remain healthy is practically advantageous for yourself and society at large. Algorithmic profiling unveiling your near-obsessive search for vacation deals may lead you to see that you need a break and help to make better decisions about your work-life balance. However, while there is no doubt that technology can provide large quantities of personal information, its quality regarding significance and accuracy is often lacking. Given that many personal information technologies also involve high costs, in terms of time and financial investments as well as a loss of privacy, we should carefully assess whether those resources could be invested elsewhere with an increased contribution to self-knowledge at lower costs.

### Difficulties for critical engagement

Technology provides impersonal self-knowledge. But, as mentioned above, you can take an agentive, critical stance towards this impersonal self-knowledge by interpreting it, embracing or rejecting it, and being actively engaged in how it defines yourself. However, when pursuing self-knowledge through technology, multiple factors hamper the employment of these faculties of critical self-knowledge.

The technologically sourced personal information is often hard to understand and assess. The hardware and the algorithms used to generate personal profiles and bioinformation is usually company property and not accessible. Moreover, even if the information were accessible, it would often require a lot of expertise to understand it. What the electrodes of a DTC neurotechnological device measure and how this translates, for instance, to stress levels is difficult to understand for a layperson. Similarly, the process of how recidivism risk is algorithmically calculated is not accessible to the public and is, at least in some cases, complex. To engage critically with the impersonal self-knowledge provided by those technologies, i.e., to make up your mind about it, ascribe meaning to it, interpret it, or avow it you have to sufficiently understand it. The lack of transparency makes it particularly hard to assess the value of personal information and to know whether and in which situation to trust personal information technology.

The fact that there is often an unjustified hype for novel technologies amplifies this effect. Inherent uncertainties and inaccuracies are often not sufficiently communicated and are overlooked in the initial enthusiasm. A reason for the tenacity of this hype is that the user of those technologies – be it the person who tracks his sleep or the justice system – is often not or not adequately guided by specialists. For instance, brain imaging used to be conducted by specialized medical professionals, but with DTC neurotechnology everyone can record the electromagnetic waves of their brains. This comes at the disadvantage that the results are more likely to be misinterpreted and trusted more than would be warranted.^7^

A further difficulty for critical engagement is that the claims technology makes about us are often of statistical nature. Humans are notoriously bad at understanding probabilities (Tversky & Kahneman, 1974). On top of that, it is hard to take a stance on statistical information and actively decide whether and how it defines you. How do you reject or embrace, give personal meaning to, or enact the information that you are *likely* to be outgoing, want to have children soon, develop Alzheimer’s disease, or be a reoffender? In some sense, statistical information is not about *you* but about an abstracted model of you. This model only takes certain parameters into account, e.g., your genetic data or your online purchase profile. All the other features of yourself determine where you personally fall on this likelihood spectrum – whether you are part of the 40% of people who fit this model who will develop Alzheimer’s disease or not. Usually, it is unclear how your other features factor into, e.g., whether you will develop Alzheimer’s. Therefore, it remains uncertain what a statistical piece of information means for *you personally*. Particularly if the likelihood is not very high or low it can be hard to draw relevant conclusions from statistical information.

Some categorizations, notably the groups created by recommender systems or for targeted advertising, can also be alienating. They may appear alien and disconnected from your lived experience because they do not have a corresponding group identity in real life. Should you feel affiliated with the group of novelty-sweater buyers in which you have been placed? It becomes difficult to engage critically with those categories because it is hard to understand what they mean, evaluate the appropriate reaction, or challenge them (de Vries, 2010, Leuenberger, forthcoming-a).

Furthermore, personal information technology can lead us to question the degree of control we have over who we are. It encourages an image of identity as passively discovered rather than actively and dynamically created. Particularly neuroinformation can lead to the false assumption that because something is “ingrained in the brain” there is not much to do about it. The growing technologically sourced impersonal knowledge available to us can make it seem like everything about us has been measured and determined. Thereby, it limits options for creative self-definition – for who you can take yourself to be (Engelmann et al., 2022; Floridi, 2011, Leuenberger, forthcoming-b).

By restricting opportunities for self-definition, those technologies can also be considered as limiting privacy. Following Agre’s account, a right to privacy can be understood as “the freedom from unreasonable constraints on the construction of one’s own identity. This idea is appealing for several reasons: it goes well beyond the static conception of privacy as a right to seclusion or secrecy, it explains why people wish to control personal information, and it promises detailed guidance about what kinds of control they might wish to have.” (Agre, 1998, 7) Recommender systems can further reinforce this effect of restricted self-creation by keeping you within your individual bubbles of news, movies, products, and political opinions you like. Thereby, your identity is less likely to be challenged and you are less inclined to actively and critically affirm or change your beliefs and preferences.

The difficulty of engaging critically with technologically sourced impersonal self-knowledge comes with disadvantages. As discussed above, this critical, agentive stance can turn trivial into substantial self-knowledge. Through interpretation, ascription of meaning, and by forming an attitude towards trivial, impersonal self-knowledge, it can become substantial. The nature of technologically sourced personal information complicates this critical engagement that can enrich trivial, impersonal self-knowledge. Moreover, critical self-knowledge is important for agency and responsibility. If your identity is largely construed of passively discovered instead of actively and critically chosen and created elements, you may take less responsibility for it and decrease your agency over it.

However, there is also value in approaching elements of one’s identity as impersonal self-knowledge instead of establishing a more active relation. As Levy argues, addicts can benefit from reaffirming their addiction in terms of impersonal self-knowledge instead of critically engaging with it (Levy, 2016). It can help to stand by one’s convictions because one does not reopen courses of action (e.g., to relapse) to negotiation and one can learn how to employ indirect control.

In some cases, technological self-knowledge can also have the opposite effect and foster one’s active self-constitution and critical self-knowledge. Particularly health and activity trackers encourage self-management and self-improvement. The data of health-tracking apps is often collected to help users to reach a goal and it is presented in a way that motivates them to reach that goal. They nudge them to engage in behavior they avowed and to reach goals they set for themselves. Thereby, they can support us in “making true what is claimed to be true” (Bransen 2015, 316) – i.e., in making our decisions and resolutions come true.

### Relational dimensions

As discussed above, knowing yourself includes knowing “your place in the world”, how you relate to others, how they see you, and how you fit into groups and institutions. Others provide and restrict your opportunities, define concepts through which you can define and constitute yourself, and give feedback on how you appear to them. How you appear from an external perspective and are constrained by it is part of who you are – knowing yourself entails knowing about this. Those external perspectives are not just other people but increasingly also technologies that measure you, create profiles, and make or influence decisions about you.

Particularly in terms of practical value, this kind of self-knowledge can be very important. Even if the algorithm is flawed and biased, it is still good to know what it says about you, especially if it decides about your job opportunities or sentencing. You may not learn from an algorithm whether you are actually suitable for a job, but you learn whether you are the kind of person who is considered suitable and will get the job. One might be tempted to think that a flawed algorithm that falsely considers someone to be unsuitable for a job or likely to reoffend says more about this society, the technology, and its developers, than about the individual itself and has nothing to do with self-knowledge. But it can reveal something about the role or place they have been assigned in the world and the opportunities and restrictions they face. Insight into biased algorithms can reveal the common social biases they reproduce (which individuals already face in other situations) as well as novel biased constraints and opportunities the algorithm imposes. Both shape the scope of action we are offered and who others let us be. For instance, the fact that only 6% of Facebook users who got to see a job advertisement for a mechanic are women (GlobalWitness, 2023) is indicative of the roles women are meant to take in this job market, which jobs are considered suitable for them, and that they still face obstacles when trying to pursue a career as a mechanic. In this sense, even an unreliable and biased technology can be a basis for a justified belief in terms of relational self-knowledge.

For the pursuit of self-knowledge through technology, this means that knowing how an algorithm is profiling you and which parameters go into creating personal profiles and influencing decisions can be relevant self-knowledge. Of course, there is no need to know what everyone and every algorithm thinks of you but knowledge about those that shape your opportunities, scope of action, and important self-defining concepts is substantial. The value of self-knowledge speaks in favor of transparency and accessibility of personal information technology. Particularly decision-making and -supporting algorithms should be more transparent in how they operate and which parameters they take into account. All too often those who are subject to algorithmic decision-making have no way of knowing how those algorithms operate or even whether an algorithm was involved in the decision. At least in theory, technology has the potential to be more transparent than human decision-making and profiling, which often involves barely accessible unconscious biases and tacit knowledge.

Bioinformation technologies and digital profiling are also involved in indirect relational influences on the self. As I have argued elsewhere (Leuenberger, forthcoming-b), personal information technology can influence which characteristics (e.g., character, values, emotions, abilities, aptitudes) are considered as meaningfully defining a person as well as how such meaningful personal characteristics are defined and measured. The widespread availability of genetic ancestry testing has turned genetic ancestry into a relevant self-defining feature for many people. Many identify with their heritage, try to connect with the respective culture, and consider it an important aspect of their identity. Personal information technologies can also set new standards for how certain characteristics are defined and measured. Heritage is now more likely to be defined in terms of genetics instead of upbringing. Moreover, technology has the power to shape what you consider meaningful and important aspects of yourself, simply by shifting your focus. According to a slogan of the Quantified Self Movement: w*hat gets measured gets managed*. By measuring parameters of your physical health or your brain waves, you are more likely to invest time and energy to optimize *those* features. Labels used by recommender systems and for targeted advertisingement also perpetuate the identified characteristics. They make users more likely to be interested in, like, and consume the products identified to suit them based on their profiles because they recommend and advertise them. Thereby, they can foster less meaningful but more commercially lucrative identity aspects.

Especially if the use of a technology is widespread, the labels and concepts used to characterize people can shape how you define yourself and others and ultimately who you are. Developing a self-conception and conceptualizing your mental states can have self-transforming effects because your conceptual capacities influence your experiences as well as your actions (Moran, 2001). The categories and concepts created and applied by different technologies can influence your conceptual space positively or negatively. In the negative case, those identity labels and concepts can be harmful, bigoted, or promote stereotypes. They can, for instance, damage self-confidence by labelling someone with a socially devalued trait or enforce sexist stereotypes when an algorithm assumes someone is female because they like typically female hobbies. In light of the self-defining powers of personal information technology, we should ensure that the categories and labels used to characterize us avoid identity harms and that users are able to understand, challenge, and potentially correct them. Moreover, it is important to be particularly careful when correlating identity labels with neurodata or genetic data because it could contribute to an essentialization of groups of people and promote the idea that certain characteristics are natural or unchangeable for certain groups (Hauskeller, 2004; Postan, 2022).

## Conclusion

Self-knowledge is a good and we have practical and moral reasons to pursue it. Technology has the potential to give us more of it – if we use it right. It can give us unique insights into our bodies, behavior, and mental states and it can convince us to believe in inaccurate and biased information. Both the benefits and the harms of those technologies are not distributed equally and justly among people. The vast range of personal information bioinformation technologies and algorithmic profiling provide can broaden our self-knowledge but various features of those technologies can make it particularly hard to engage critically and agentive with this information. Thereby, they can foster the idea of identity as discovered instead of actively created. Because we are also increasingly defined by technology – by the opportunities, constraints, and concepts it creates – knowing ourselves can entail knowing the technologies that shape us and how they characterize us. We should ensure that technologies that provide us with information about ourselves not only serve commercial interests but our interest in the pursuit of self-knowledge through accurate, transparent, inclusive, and accessible personal information.

## Footnotes

1 Some philosophers have argued for a connection between epistemological and ethical considerations regarding self-knowledge (Frankfurt 1971; Taylor 1989) (I would like to thank the anonymous reviewer for raising this point). This connection has, however, rarely been used to uncover issues in applied ethics.

2 However, self-tracking has also been discussed to commonly lead to experiences of self-related uncertainty (Bergroth 2019).

3 However, the insights companies offer to their users into why they are shown certain ads or which characteristics have been attributed to them have been criticized as often incomplete, sometimes misleading, and vague (I would like to thank the anonymous reviewer for raising this point). True targeting attributes may be obfuscated in the explanations companies offer for why users see ads by only referring to common attributes selected for advertising while hiding rare (and potentially sensitive) ones (Andreou et al., 2018).

4 This is not to deny that self-ignorance can also have pragmatic value in some cases.

5 The arguments that rationality requires self-knowledge usually refer to self-knowledge as knowing one’s beliefs, which can, at least in some cases, be trivial knowledge (Burge 1996; Shoemaker 1988).

6 I would like to thank the anonymous reviewer for raising this point.

7 Note that we also tend to have an unwarranted degree of trust in other sources self-knowledge, for instance in our memories.
